# Daily variations of gut microbial translocation markers in ART-treated HIV-infected people

**DOI:** 10.1186/s12981-020-00273-4

**Published:** 2020-05-12

**Authors:** Jing Ouyang, Stéphane Isnard, John Lin, Brandon Fombuena, Debashree Chatterjee, Tomas Raul Wiche Salinas, Delphine Planas, Amélie Cattin, Augustine Fert, Etiene Moreira Gabriel, Laurence Raymond Marchand, Yonglong Zhang, Malcolm Finkelman, Yaokai Chen, Daniel E. Kaufmann, Nicolas Cermakian, Petronela Ancuta, Jean-Pierre Routy

**Affiliations:** 1 Infectious Diseases and Immunity in Global Health Program, Research Institute, McGill University Health Centre, 1001 Blvd Décarie, Montreal, QC Canada; 2 Chronic Viral Illness Service, McGill University Health Centre, Montreal, QC Canada; 3 Chongqing Public Health Medical Center, Chongqing, China; 4 Department of Microbiology and Immunology, McGill University, Montreal, QC Canada; 5 Centre de Recherche du Centre Hospitalier de l’ Université de Montréal (CRCHUM), Montréal, QC Canada; 6 Département de Microbiologie, Infectiologie et Immunologie, Faculté de Médecine, Université de Montréal, Montréal, QC Canada; 7 Associates of Cape Cod Inc, Falmouth, MA USA; 8 Douglas Research Centre, Department of Psychiatry, McGill University, Montréal, QC Canada; 9 Division of Hematology, McGill University Health Centre, Montreal, QC Canada

**Keywords:** Daily variation, Microbial translocation, (1 → 3)-β-D-Glucan, Gut damage, HIV

## Abstract

**Background:**

Increased intestinal barrier permeability and subsequent gut microbial translocation are significant contributors to inflammatory non-AIDS comorbidities in people living with HIV (PLWH). Evidence in animal models have shown that markers of intestinal permeability and microbial translocation vary over the course of the day and are affected by food intake and circadian rhythms. However, daily variations of these markers are not characterized yet in PLWH. Herein, we assessed the variation of these markers over 24 h in PLWH receiving antiretroviral therapy (ART) in a well-controlled environment.

**Methods:**

As in Canada, PLWH are predominantly men and the majority of them are now over 50 years old, we selected 11 men over 50 receiving ART with undetectable viremia for more than 3 years in this pilot study. Blood samples were collected every 4 h over 24 h before snacks/meals from 8:00 in the morning to 8:00 the next day. All participants consumed similar meals at set times, and had a comparable amount of sleep, physical exercise and light exposure. Plasma levels of bacterial lipopolysaccharide (LPS) and fungal (1→3)-β-D-Glucan (BDG) translocation markers, along with markers of intestinal damage fatty acid binding protein (I-FABP) and regenerating islet-derived protein-3α (REG3α) were assessed by ELISA or the fungitell assay.

**Results:**

Participants had a median age of 57 years old (range 50 to 63). Plasma levels of BDG and REG3α did not vary significantly over the course of the study. In contrast, a significant increase of LPS was detected between 12:00 and 16:00 (Z-score: − 1.15 ± 0.18 vs 0.16 ± 0.15, p = 0.02), and between 12:00 and 24:00 (− 1.15 ± 0.18 vs 0.89 ± 0.26, p < 0.001). The plasma levels of I-FABP at 16:00 (− 0.92 ± 0.09) were also significantly lower, compared to 8:00 the first day (0.48 ± 0.26, p = 0.002), 4:00 (0.73 ± 0.27, p < 0.001) or 8:00 on secondary day (0.88 ± 0.27, p < 0.001).

**Conclusions:**

Conversely to the fungal translocation marker BDG and the gut damage marker REG3α, time of blood collection matters for the proper evaluation for LPS and I-FABP as markers for the risk of inflammatory non-AIDS co-morbidities. These insights are instrumental for orienting clinical investigations in PLWH.

## Background

The gastrointestinal tract is a distinctive tissue with physical, biological and immunological barriers, allowing nutrient absorption while preventing the translocation of microbes and their products. HIV infection is associated with modification of the gut microbiota, disruption of the gut epithelial barrier, and increased intestinal permeability [1–4]. In contrast to the global health improvement occurring in people living with HIV (PLWH) receiving antiretroviral therapy (ART), gut damage persists and translocation of microbial products from the gut lumen into the circulation contributes to inflammatory non-AIDS comorbidities [5]. Microbial translocation is one of the main drivers for the development of such comorbidities including cardiovascular disease, depression and cancer in ART-treated PLWH [6–10].

Measurement of microbial translocation plasma markers have been frequently performed in studies evaluating therapeutic interventions and assessing the risk of developing non-AIDS co-morbidities [11–27]. Circulating levels of lipopolysaccharide (LPS) are commonly measured to assess the level of bacterial translocation. LPS is a bacterial cell wall polysaccharide and is a well-known inducer of innate immune activation [13]. Besides bacterial translocation, there is increasing awareness regarding fungal translocation [24, 28–30]. Fungi contribute greatly to opportunistic infections in PLWH, including *Pneumocystis jirovecii* in the respiratory tract and *Candida albicans* in the gastrointestinal tract [31]. (1 → 3)-β-D-Glucan (BDG) is a major component of most fungal cell walls and serves as a potent pathogen-associated molecular pattern (PAMP) in triggering antifungal immunity [32]. Circulating BDG is currently used for the clinical diagnosis of *Candida*, *Aspergillus*, and *Pneumocystis jirovecii* invasive infections [33]. Recently, we and others have found that plasma levels of BDG are associated with epithelial gut damage and risk of developing inflammatory non-AIDS comorbidities in PLWH without invasive fungal infection (IFI) [24, 25, 28, 29, 33–36]. We have also shown that plasma BDG levels are associated with reduced expression of Dectin-1 and NKp30 on monocytes and NK cells respectively, indicating direct cellular activation and inflammation by BDG. Circulating BDG contributes to low grade inflammation [28, 37] and may enhance trained immunity at the epigenetic level [38, 39]. Therefore, assessment of BDG levels may be useful in predicting the risk of PLWH to develop non-AIDS comorbidities [24–26].

Circulating intestinal fatty acid binding protein (I-FABP) and regenerating islet-derived protein-3α (REG3α) are two validated gut damage markers in PLWH [40, 41]. I-FABP, an intracellular protein constitutively expressed in enterocytes, is released upon cell death and subsequently detected in the blood in inflammatory bowel diseases (IBD) and HIV infection [42, 43]. REG3α, an antimicrobial peptide secreted by intestinal Paneth cells into the gut lumen and upon gut damage, translocates into the blood [41]. We observed that REG3α plasma levels were correlated with HIV disease progression, microbial translocation and immune activation in PLWH [41].

As levels of gut damage and microbial translocation markers are low in healthy people and significantly elevated in PLWH [44, 45], knowing their daily variations could improve clinical care and research. Herein, we assessed the variation of the microbial translocation markers, LPS and BDG, and the gut damage markers, I-FABP and REG3α, over the course of 24 h in ART-treated PLWH in a well-controlled environment.

## Methods

### Participants and study design

**Fig. 1:**
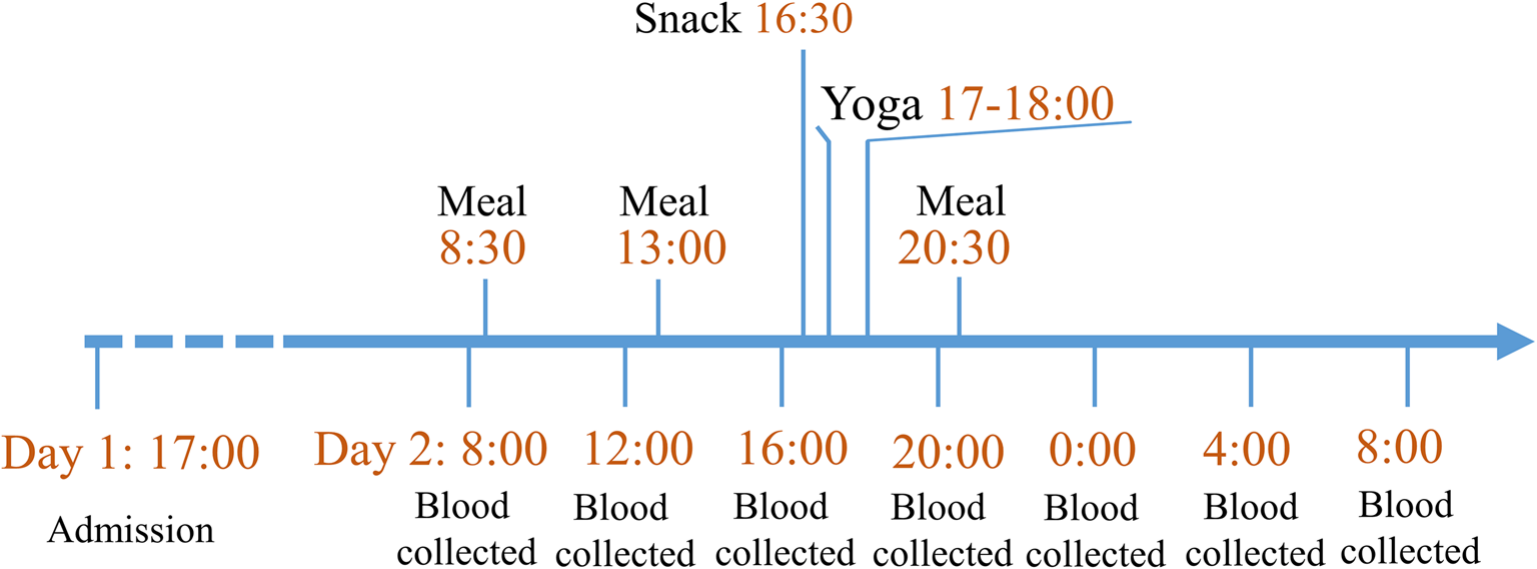
Study timeline

In this pilot study, 11 men were recruited as they represent the population most affected by HIV in Canada. Inclusion criteria included men over the age of 50, receiving ART with undetectable viremia for more than 3 years. Participants with opportunistic infections (including fungal infections), co-infection with hepatitis B or C, chronic colitis or any other acute conditions were excluded. A total of 11 participants were enrolled and hospitalized for 40 h at the 12-bed phase I clinic of the Centre Hospitalier de l’Université de Montréal, Montréal, QC, Canada. Study timeline is shown in Fig. 1. Blood samples were collected using a catheter fixed to the median cubital vein throughout their hospitalization to prevent repeated venipuncture and disturbing participants’ sleep cycles. All the participants were admitted to hospital before 17:00 of the previous day in order for all participants to have the same meal and similar amount of sleep before baseline sampling. Samples were first collected at 8:00 am, and then every 4 h until 8:00 am the next morning for a total of seven timepoints. Plasma was isolated from whole blood and frozen at − 80 °C. All participants had similar meals/snacks at set times (8:30, 13:00, 16:30 and 20:30), and had a comparable amount of sleep, physical exercise and light exposure. All participants were offered the same meals, with a balance of carbohydrates, proteins, fats and fibers. Meals did not contain mushrooms nor seaweed. BDG containing foods such as oat bran were not offered to participants. Scientific and artistic educational presentations were organized as part of a knowledge transfer and exchange with participants and research nurses. All participants agreed to take part in a 60 min low-intensity yoga session in the afternoon. Neither alcoholic beverages nor recreational drugs were permitted during the time of hospitalization.

### Laboratory measurements

Plasma HIV-1 p24 antigen/antibody and confirmatory Western blot tests were used to confirm HIV-infection, as previously reported [28]. Quantification of plasma viral load (VL) was done using the Abbott Real-Time HIV-1 assay (Abbott Laboratories, Abbott Park, Illinois, USA). CD4 and CD8 T-cell counts were measured using flow cytometry. LPS was quantified using a human lipopolysaccharide enzyme-linked immunosorbent assay (ELISA) (Cusabio, Wuhan, China) to avoid cross-reactivity with BDG in existing limulus amebocyte lysate (LAL) assays [28]. Plasma BDG level was measured by the Fungitell^®^ LAL assay (Associates of Cape Cod, Inc., East Falmouth, MA, USA) [28]. I-FABP and REG3α were quantified in plasma using ELISA (Hycult Biotech, Uden, Netherlands and R&D systems, Oakville, ON, Canada, respectively) [41].

### Statistical analyses

Statistical analyses were conducted using GraphPad Prism 6.0 (La Jolla, CA, USA). In order to analyze the time variation of parameters while accounting for inter-participant variability, the individual raw data were converted to Z scores as previously described [46]. Z-scores were calculated by normalizing each datapoint to the mean of the seven timepoints and standard deviation (SD) of the individual according to the formula: (datapoint-mean)/SD. Comparisons were conducted between timepoints using non-parametric Kruskal–Wallis test with Dunn’s post hoc test. An α-level of 5% was used for statistical significance.

## Results

### Study participant characteristics

Participant characteristics were summarized in Table 1. All participants were male, with a median age of 57 years (range 50 to 63). Participants received ART for a median of 17 years (range 13 to 22). Plasma VL of all participants were below the level of detection (< 20 copies/ml). Median CD4 T-cell count was 606 (311–1082) cells/µl and CD8 T-cell count was 613 (331–1425) cells/µl.

**Table 1:** Participant characteristics (n = 11)

ID	Age	Body mass index(kg/m^2^)	CD4 count (cells/µL)	CD8 counts (cells/µL)	ART duration (years)	Viral load	Current ART medication
1	60	27.8	602	1321	10	Undetectable	Emtricitabine, TDF, raltegravir
2	52	27.1	491	613	21	Undetectable	Emtricitabine, TDF, raltegravir
3	57	28.4	606	855	12	Undetectable	Emtricitabine, TDF, raltegravir
4	57	32.9	846	901	22	Undetectable	Emtricitabine, TDF, darunavir, cobicistat
5	57	24.9	410	924	31	Undetectable	Emtricitabine, TDF, efavirenz
6	63	27.7	667	553	15	Undetectable	Abacavir, dolutegravir, lamivudine
7	50	34.9	379	498	19	Undetectable	Emtricitabine, TDF, raltegravir
8	58	26.1	311	331	21	Undetectable	Emtricitabine, TDF, elvitegravir, cobicistat
9	57	24.6	800	597	13	Undetectable	Abacavir, dolutegravir, lamivudine
10	58	32.1	675	494	13	Undetectable	Abacavir, dolutegravir, lamivudine
11	54	23.9	1082	1425	17	Undetectable	Lamivudine, abacavir, raltegravir

TDF, tenofovir disoproxil fumarate

### Daily variation of the microbial translocation markers LPS and BDG

**Fig. 2:**
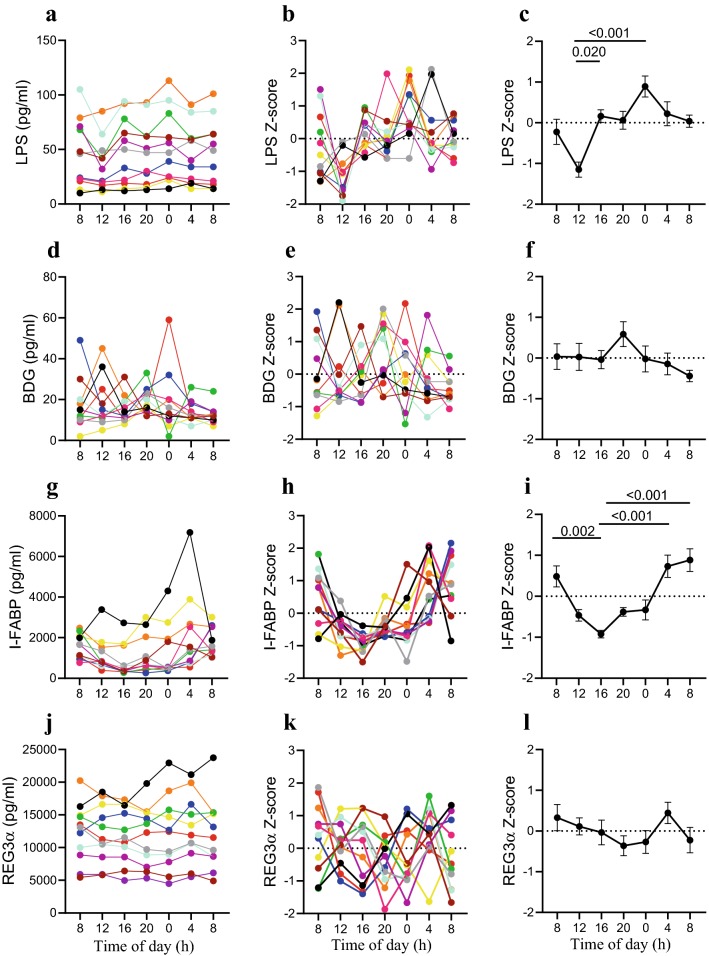
Daily variation of gut damage and translocation markers. Plasma levels of Lipopolysaccharide (LPS) (**a**–**c**, p < 0.001), (1 → 3)-β-D-Glucan (BDG) (**d**–**f**, p = 0.261), Intestinal fatty acid binding protein (I-FABP) (**g**–**i**, p < 0.001) or Regenerating islet-derived protein 3 α (REG3α) (**j**–**l**, p = 0.570). In figure **a**–**k**, different colors represent different participants: red (ID 1); orange (ID 2); yellow (ID 3); green (ID 4); blue (ID 5); cyan (ID 6); purple (ID 7); pink (ID 8); gray (ID 9); black (ID 10); brown (ID 11). Mean ± standard error of the mean (SEM) of the Z-score are shown in (**c**, **f**, **i**, **l**). Friedman test

Data are presented as raw concentration of markers at each timepoint (left column), individual participant’s Z-score (middle column) and average Z-score (right column). Comparison between time points showed that LPS levels varied significantly over time (p < 0.001) and tended to decrease between 8:00 to 12:00 (Z-score − 0.22 ± 0.31 vs. − 1.15 ± 0.18), without reaching statistical significance (Fig. 2c). A significant increase of LPS was observed from 12:00 to 16:00 (Z-score of − 1.15 ± 0.18 vs. 0.16 ± 0.15, p = 0.02) (Fig. 2c). Similarly, a difference was also noticed between 12:00 and 24:00 (Z-score of − 1.15 ± 0.18 vs. 0.89 ± 0.26, p < 0.001) (Fig. 2c). At 8:00 on the second day, levels of LPS were comparable to levels observed at 8:00 on the preceding day (Fig. 2c).

Conversely, levels of BDG did not vary significantly over the course of the study (Fig. 2d–f, p = 0.261).

### Daily variation of the gut damage markers I-FABP and REG3α

Over the course of the study, I-FABP levels varied significantly (p < 0.001). I-FABP levels decreased from 8:00 to 16:00 with a Z-score 0.48 ± 0.26 vs. − 0.92 ± 0.09 (p = 0.002) (Fig. 2i). After 16:00, levels of I-FABP increased [Z-score of 0.73 ± 0.27 at 4:00 (p < 0.001) and 0.88 ± 0.27 at 8:00 (p < 0.001)]. Similar levels of I-FABP were observed at 8:00 of the first day and 8:00 of the second day (p > 0.05) (Fig. 2g).

Levels of the gut damage marker REG3α did not vary significantly over the course of the study (Fig. 2J–l, p = 0.570).

## Discussion

We observed a daily variation of plasma I-FABP with a median difference of 2060 pg/ml between the highest and lowest values, representing a 4.2-fold intraday variation. The median plasma LPS range between highest and lowest values was 18 pg/mL, representing a 1.6-fold intraday change. Such variations might explain the breadth of I-FABP and LPS levels observed in other ART-treated PLWH studies where time of sampling was not documented or considered [13, 28, 41]. Previous work in mouse models has shown a postprandial increase in LPS levels [47]. We observed a clinically relevant decrease of LPS after breakfast and an increase after dinner which might be explained by natural variations in circadian rhythm [48, 49]. These results suggest that sampling LPS from fasting PLWH might decrease variation throughout the day. Similarly, I-FABP was subject to daily variations with the lowest level at 16:00 and highest at 4:00-8:00. However, plasma levels of BDG and REG3α showed no significant variation and were not affected by food intake, time of sampling, or day/night shifts. These findings further validate the use of BDG and REG3α as markers of microbial translocation and gut damage, respectively in ART-treated PLWH.

Translocation of bacterial and fungal products is driven by epithelial gut damage and depletion of intestinal CD4-T cells and contributes to immune activation in HIV [35, 50]. Clinical studies commonly use circulating I-FABP to evaluate gut damage as a measure of enterocyte cell lysis. However, in the absence of enterocyte lysis, I-FABP poorly correlates with microbial translocation [41]. Our results show that circulating I-FABP levels varied greatly throughout the course of a day, which limits its value as a marker of gut damage, since it is dependent on the time of sampling and fasting status. In contrast to I-FABP, REG3α appeared stable over the course of 24 h. Therefore, our results and previous work favor REG3α as a reliable gut damage marker independent of sampling time and food intake in PLWH [41].

LPS is a bacterial translocation marker, responsible for chronic immune activation in HIV-infected patients [51]. However, increasing evidence indicates that diet and food intake affect the plasma level of LPS in mouse and human models. Cani et al. [47] first reported in 2007 that plasma levels of LPS increased after feeding mice with a high-fat diet. Furthermore, López-Moreno et al. [52] reported that the consumption of diet rich in saturated fat increased plasma levels of LPS which in turn, increase the postprandial inflammatory response in subjects with metabolic syndrome. Our results also indicated that food intake was associated with an increase in plasma level of LPS in ART-treated PLWH up to 4 hours after lunch and supper. Although the underlying mechanism is unclear, it may be related to changes in microbiota composition, increases in the proportion of LPS producing Gram-negative bacteria in the presence of nutrients [53]. LPS detoxification by the intestinal alkaline phosphatase [54], or fat intake promoting gut translocation of LPS [47, 52]. Therefore, monitoring LPS levels in PLWH should take into account feeding state and time of specimen acquisition.

Unlike LPS, we showed that the fungal translocation marker BDG is stable throughout the day and independent of food intake. BDG can be found in food such as mushroom and seaweed [55, 56]. Interestingly, Hashimoto et al. reported that serum BDG value was elevated due to intake of seaweed in a hematopoietic stem cell transplant recipient [57]. However, the elevation of BDG may have been linked to gut damage with increased intestinal permeability during acute graft-versus-host disease (GVHD). Nevertheless, our results showed that BDG is a reliable marker for fungal translocation in ART-treated PLWH. The food provided in our study did not comprise mushroom, seaweed or other material rich of BDG. Thus, further studies need be conducted in order to study the effects of BDG rich food on its plasma level.

We acknowledge that our study presents some limitations as we did not study the underlying mechanism of daily variation of I-FABP or LPS levels. Daily changes in the external environment may also influence those markers and studies have identified the molecular underpinnings of oscillations in circadian clock gene expression occurring over the 24-hour day [49]. Our study population only included a small sample size of male participants over the age of 50, therefore younger participants and inclusion of female participants will be needed to generalize study findings to a larger population, compared to a HIV-uninfected control group.

## Conclusion

To our knowledge, we are the first to report the daily variation of different microbial translocation with gut damage markers in ART-treated PLWH. We showed that conversely to I-FABP and LPS, plasma levels of REG3α and BDG can be considered as reliable markers of gut damage and fungal translocation respectively, and are not influenced by food intake, time of sampling, or day/night shifts. Our findings provide reference for clinical research, focusing on the assessment of blood markers of gut damage and microbial translocation. The clinical implications of the daily variation of these markers should be assessed in larger cohorts of ART-treated PLWH, including male and female participants from different ages and ethnicity.

## Data Availability

The datasets during and/or analysed during the current study available from the corresponding author on reasonable request.

## Abbreviations

ART: Antiretroviral therapy
LPS: Lipopolysaccharide
BDG: (1 → 3)-β-D-Glucan
GVHD: Graft-versus-host disease
LAL: Limulus amebocyte lysate
IBD: Inflammatory bowel diseases
I-FABP: Intestinal fatty acid binding protein
IFI: Invasive fungal infection
PAMP: Pathogen-associated molecular pattern
PLWH: people living with HIV
REG3α: Regenerating islet-derived protein-3α
SD: Standard deviation
VL: Viral load
